# Over-activation of TRPM2 ion channel accelerates blood-spinal cord barrier destruction in diabetes combined with spinal cord injury rat

**DOI:** 10.7150/ijbs.80672

**Published:** 2023-05-08

**Authors:** Susu Zhang, Jiaxin Zhao, Man Wu, Yongxiu Zhou, Xuejuan Wu, Anyu Du, Yibing Tao, Shanshan Huang, Shufang Cai, Mei Zhou, Tao Wei, Yanren Zhang, Ling Xie, Yanqing Wu, Jian Xiao

**Affiliations:** 1 Department of Wound Healing, The First Affiliated Hospital and School of Pharmaceutical Sciences, Wenzhou Medical University, Wenzhou, Zhejiang, 325000, China.; 2 The Institute of Life Sciences, Engineering Laboratory of Zhejiang Province for Pharmaceutical Development of Growth Factors, Wenzhou University, Wenzhou, 325035, China.

**Keywords:** Spinal cord injury (SCI), Diabetes, Endothelial cells (ECs), Transient receptor potential melastatin 2 (TRPM2), Reactive oxygen species (ROS)

## Abstract

Spinal cord injury (SCI) is a devastating neurological disorder that often results in loss of motor and sensory function. Diabetes facilitates the blood-spinal cord barrier (BSCB) destruction and aggravates SCI recovery. However, the molecular mechanism underlying it is still unclear. Our study has focused on transient receptor potential melastatin 2 (TRPM2) channel and investigated its regulatory role on integrity and function of BSCB in diabetes combined with SCI rat. We have confirmed that diabetes is obviously not conductive to SCI recovery through accelerates BSCB destruction. Endothelial cells (ECs) are the important component of BSCB. It was observed that diabetes significantly worsens mitochondrial dysfunction and triggers excessive apoptosis of ECs in spinal cord from SCI rat. Moreover, diabetes impeded neovascularization in spinal cord from SCI rat with decreases of VEGF and ANG1. TRPM2 acts as a cellular sensor of ROS. Our mechanistic studies showed that diabetes significantly induces elevated ROS level to activate TRPM2 ion channel of ECs. Then, TRPM2 channel mediated the Ca^2+^ influx and subsequently activated p-CaMKII/eNOS pathway, and which in turn triggered the ROS production. Consequently, over-activation of TRPM2 ion channel results in excessive apoptosis and weaker angiogenesis during SCI recovery. Inhibition of TRPM2 with 2-Aminoethyl diphenylborinate (2-APB) or TRPM2 siRNA will ameliorate the apoptosis of ECs and promote angiogenesis, subsequently enhance BSCB integrity and improve the locomotor function recovery of diabetes combined with SCI rat. In conclusion, TRPM2 channel may be a key target for the treatment of diabetes combined with SCI rat.

## Introduction

Traumatic spinal cord injury (SCI) is one of the major causes of public health problems worldwide, which causes series of physical and psychological effects, such as quadriplegia and paraplegia 1, 2. SCI results in BSCB destruction during primary and secondary injury, and leads to a range of deleterious effects, including inflammation, oxidative stress and mitochondrial dysfunction, which ultimately contributes to apoptosis of endothelial cells (ECs), and then inhibits vessel regeneration and neural functional recovery 3, 4. Due to bedridden, SCI patients often exhibit symptoms of weight gain and altered metabolism 5. Thus, SCI patients have also been reported to be at higher risk for hyperlipidemia, diabetes, metabolic syndrome and coronary artery disease than the general population 6. In turn, diabetes hinders the recovery of SCI 7. Recently, the proportion of diabetes combined with SCI patients is increasing. As it is well known that diabetes could not only induce oxidative stress, inflammation and mitochondrial dysfunction in ECs, but is also not conductive to angiogenesis 8, 9. In our previous study, we have also confirmed that diabetes induces the more severe BSCB destruction after SCI 10, 11. However, the specific mechanism of diabetes-mediated aggravating BSCB destruction is still unknown.

BSCB destruction induces the inflammation and ischemia, which is the main causes of secondary injury. Promoting BSCB repair is an important task for promoting SCI recovery. Neurovascular unit in spinal cord is composed of ECs, vascular basement membrane, pericytes, glial cells and adjacent neurons, which is the most basic structure for maintaining BSCB integrity in spinal cord. Among them, ECs are the main components of BSCB, which forms the tight structural barriers by tight junction (TJ) proteins and adhesion junction (AJ) proteins, and effectively blocks the intercellular movement of macromolecules. Therefore, protecting ECs is important for promoting BSCB repair after SCI. ECs dysfunction is the earliest and most fundamental pathological change under hyperglycemic condition. A large amount of evidence has shown that diabetes can induce the excessive superoxide, trigger DNA damage, and thereby promote vascular cell damage 12, 13. We hypothesized that diabetes-mediated ECs damage may be an essential factor for diabetes aggravating BSCB destruction after SCI. Our previous study had demonstrated that compared with SCI rat, diabetes combined with SCI rat shows the increased penetration of evans blue (EB) dye, more severe BSCB destruction, and loss of ECs and pericytes at day 1 post SCI 11.

Continuous and stable blood supply is an important guarantee for axon regeneration and nerve repair after SCI. After injury, the injured area is exposed in an ischemic and hypoxic microenvironment. Thus, vascular regeneration and functional reconstruction are essential for subsequent nerve repair after SCI. Hypoxia inducible factor-1α (HIF-1α), vascular endothelial growth factor (VEGF) and angiopoietin 1 (ANG1) are the important regulators during angiogenesis. Among them, HIF-1α is a classical transcription factor that widely exists in mammals and humans. HIF-1α responses to the hypoxic signaling and induces the expression of hypoxia gene to regulate the dynamic balance of cellular oxygen content 14. VEGFA is a major pro-angiogenic factor that promotes the proliferation and migration of ECs. VEGFA expression is strongly dependent on the presence of HIF-1α 14, 15. As a natural antagonist of ANG2, ANG1 promotes the survival of vascular ECs, the formation and maintenance of blood vessels 16, 17. Moreover, ANG1 can also switch the inflammatory state of vascular from an active state to a quiescent state, which is particularly important for healing and repair after injury 18.

Imbalance of intracellular ion homeostasis is a common mechanism of oxidative stress-mediated cell death. Transient receptor potential melastatin 2 (TRPM2) is an intracellular ADP-ribose (ADPR)-gated non-selective Ca^2+^ ion channel. TRPM2 channel is widely distributed in various cells and highly sensitive to excessive ROS. Current studies suggest that H_2_O_2_-triggered TRPM2 channel activation is indirectly mediated through the production of ADPR by nicotinamide adenylate dinucleotide (NAD). Activation of TRPM2 channel leads to TRPM2-dependent accumulation of Zn^2+^ and Ca^2+^, which in turn triggers a large amount of ROS production 19. In addition, it is also reported that activation of TRPM2 channel leads to Ca^2+^ influx, promotes phosphorylation of RACK1, and then increases oxidase activity of NADPH 20. Excessive ROS production and elevated oxidative stress are the important caused mechanism of ECs apoptosis. Oxidative stress has been reported to promote the death of pericytes by mediating the activation of TRPM2 channel 21. Therefore, we have focused on the effect of TRPM2 ion channels on BSCB in diabetes mellitus with SCI.

Here, we build a T1D combined with SCI rat model and try to explore the regulatory role of TRPM2 ion channel on BSCB destruction during diabetes combined with SCI recovery. We have confirmed that diabetes aggravates BSCB destruction and impedes the locomotor functional recovery of SCI. For the mechanistic study, it has been demonstrated that under diabetic condition, TRPM2 mediates Ca^2+^ influx to activate calcium/calmodulin-dependent protein kinase (CaMKII)/eNOS in spinal cord after SCI, which in turn produces ROS production. Consequently, over-activation of TRPM2 ion channel results in excessive apoptosis and weaker angiogenesis during SCI recovery.

## Materials and methods

### Animals and ethics statement

A total of 160 healthy 8-week-old female Sprague-Dawley rats (200-250g) were purchased from Vital River Laboratory Animal Technology Co. Ltd (Beijing, China). The rats were housed in colony cages with a 12 h light/dark cycle under constant temperature and humidity (60%), and given free access to water and food. All experimental procedures were approved by the Laboratory Animal Ethics Committee of Wenzhou Medical University and performed in accordance with the National Institutes of Health guide for the care and use of Laboratory animals.

### The build of T1D combined with SCI rat model and treatment

Considering the long period for building T2D animal model and the high mortality of T2D mice after SCI, we chose T1D rat model to study the effect of hyperglycemia on SCI recovery. For type 1 diabetes mellitus (T1D) model, the rats were fasted for 12 h, and then intraperitoneally injected with 55 mg/kg STZ (Solarbio, China) (Figure S 1A). After 3 days, the body weight and blood glucose level of rats were tested (Figure S 1C and S 1D). Rats with the blood glucose level ≥16.7 mM were diagnosed as T1D rat model. After 2 weeks, these rats were randomly divided into 5 groups: Sham, T1D, SCI, T1D+SCI and T1D+SCI+2-APB group. For SCI surgery, the rats were carried out the laminectomy to exposed T9-T10 segment of spinal cord after anesthesia, then hit the T9-T10 segment of spinal cord with Allnes beater. The rat was regarded as the successful SCI model when the rats exhibited twitch, tail swing, hyperemia and swelling at the hit place. The blood glucose level of rats was tested after SCI surgery (Figure S 1E). For T1D+SCI+2-APB group, 3 mg/kg of 2-APB (Glpbio, USA) was intraperitioneally injected into rat daily from 1 day before SCI surgery to inhibit TRPM2 expression (Figure S 1A). Then, the rats were performed behavioral assessment and the spinal cord tissues were collected for molecular biological analysis.

### Locomotor function assessment

At 14 days post SCI, the footprint analysis and electromyography analysis of hind limb were used for assessing the locomotor functional recovery of rats. For footprint analysis, the hind limb of rats was immersed into a red dye and the fore limb were immersed into a blue dye. Then, the rats crawled on the paper to collect the footprints of limbs of rats. For electromyography of hind limb, the incubation and muscle amplitude were collected for further analysis when electrical signals from the spinal cord injury site stimulate the lower limb muscle contractions.

### Evaluation of BSCB permeability

The BSCB integrity was assessed by the degree of EB dye extravasation. 4 mL/kg of EB dye was injected into the tail vein of rat and waited 2 h for dye distribution. Then, the rats were anesthetized and transcardially perfused with 0.9% saline until the colorless fluid had outflowed from the right atrium. Then, the spinal cord tissues were fixed with 4% paraformaldehyde (PFA). The fluorescence intensity in transverse section of spinal cord was examined under a Leica fluorescent microscope (Leica-TCS SP8).

### Western blotting analysis

For western blotting, the 0.5 cm spinal cord tissue that obtained from the epicenter of lesion sites and human umbilical vein endothelial cells (HUVECs) were homogenated and lysed by lysis buffer (BOSTER, AR0101) with the presence of protease inhibitor (Beyotime, P1005). Bradford (Ab102535) was used to quantify the concentration of protein in lysates. Equivalent amounts of protein were separated on sodium dodecyl sulfate-polyacrylamide gel electrophoresis (7.5-12.5% SDS-PAGE), and then transferred onto a polyvinylidenefluoride (PVDF) membrane (Bio-Rad, 1620177). Then, the membranes were blocked with 5% skimmed milk in TBST for 2 h at room temperature, and incubated with the following primary antibodies at 4℃ overnight: TRPM2 (1:1000, NB500-242), p-CaMKII (1:1000, sc-32289), CaMKII (1:1000, sc-5306), eNOS (1:1000, sc-376751), NOX2 (1:1000, ab129068), HIF-1α (1:1000, ZEN BIO, 340462), ANG1 (1:5000, ab183701), VEGF (1:1000, sc-7269), Cleaved(C)-caspase3 (1:1000, 9664S), β-catenin (1:1000, ab32572), ZO-1 (1:1000, AF5145), Claudin-5 (1:1000, AF5216), occluding (1:1000, DF7504), P120 (1:1000, AF4684). After washed with TBST, the membranes were incubated with horseradish peroxidase-conjugated secondary antibodies for 1h at room temperature. Lastly, the signals were visualized by ChemiDicTM XRS+ ImagingSystem (Bio-Rad, USA), and quantified by Image Lab.

### Hematoxylin & eosin(H&E) staining and Nissl staining

After fixed in 4% PFA, the spinal cord tissues of rat were dehydrated, waxed and embed in paraffin. Then, 5-μm sections were dewaxed and hydrated, and stained with H&E staining kit (G1120, Solarbio) and Nissl staining solution (C0117, Beyotime). Finally, the images were observed under microscope (Nikon, Japan).

### Cell culture and treatment

HUVECs were purchased from the cell storage center of Wuhan University (Wuhan, China) and cultured in 4.5 g/L (25 mM) DMEM medium supplemented with 10% fetal bovine serum (FBS), 100 U/ml streptomycin and 100 U/ml penicillin at 37 ℃ with 5% CO_2_ and 95% air. We set a concentration gradient of 25 mM-100 mM of high glucose to stimulate HUVECs for 24 h. Using Cell Counting Kit-8 assay (CCK-8) (Dojindo, CK04), we selected 55mM glucose as high glucose that reduced the cell activity of HUVECs to about 60% when comparing with control group (Figure S 1F). In order to exclude the interference of osmotic pressure, we also used mannitol (regulating osmotic pressure to 55mM) to treat the HUVECs in control group. It was showed that the cell viability of HUVECs has no significant difference between Ctrl group and Ctrl+Mannitol group (Figure S1G). Thus, the HUVECs were co-cultured with high glucose (55 mM) + PA (0.1mM) (HG) for 24 h to mimic the diabetic environment (Figure S1 B). And then, the cells were treated with 100 μM H_2_O_2_ for 1.5 h to mimic the effect of ROS for ECs after SCI (Figure S1 B and S1 H). Additionally, TRPM2 small interfering RNA (TRPM2-siRNA) (GenePharma, China) and control RNA were pre-treated for HUVECs to inhibit TRPM2 expression for 12 h. Transmate (GenePharma, China) was used for transfection of siRNA into the HUVECs according to the manufacturer's protocol.

### Immunostaining analysis

For immunofluorescence, 1 cm spinal cord tissue was obtained from the epicenter of lesion in spinal cord of rat, and post-fixed in 4% PFA for 48 h, then embedded in paraffin. 5 μm of longitudinal or transverse sections and HUVECs fixed with 4% PFA were used for subsequent staining. After high-pressure antigen retrieval, the sections were permeabilized and blocked by 5% bovine serum albumin (BSA) (Sigma, A7030) for 30 min at 37℃, then incubated with primary antibodies at 4℃ for overnight. For immunohistochemistry, the sections were incubated with following primary antibodies: HIF-1α (1:200, ZEN BIO, 340462), ANG1 (1:200, ab183701), Cleaved-caspase 3 (1:200, 9664S) at 4℃ overnight. After washed with PBST 3 times, the sections were incubated with appropriated secondary antibodies for 1 h at 37℃, and then incubated with 3,3-diaminobenzidine (DAB) and counter-stained with hematoxylin. For immunofluorescence, the sections were incubated with following primary antibodies: TRPM2 (1:200, NB500-242), p-CaMKII (1:200, sc-32289), Claudin-5 (1:200, AF5216), CD31 (1:200, AF3628), GFAP (1:200, sc-33673), Iba1 (1:200, ab283319), NeuN (1:200, ab104224). After washed with PBST at 3 times, the sections were incubated with secondary antibodies (AlexaFluor FITC, AlexaFluor TRITC or AlexaFluor Cy5) for 1 h at 37℃. Then, the nuclei were counterstained with DAPI. Lastly, the images were captured under a Nikon ECLIPSE Ti microscope (Nikon, Japan).

### Flow cytometry

Flow cytometry was used to detect the apoptosis level of HUVECs using annexin V-propidium iodide (PI) detection kit (Beyotime, C1062S). The HUVECs were collected, and digested with trypsin, then incubated with FITC-conjugated annexin and PI according to protocol. The fluorescence of HUVECs was analyzed with a flow cytometer (Beckman Gallious). PI-negative and ANXA5-positive cells were considered as early apoptotic cells, PI-positive and ANXA5-positive cells were considered as advanced apoptotic cells. The results were quantified by FACScan (Beckman Gallious) and analyzed with FlowJo7.6 software.

### Tube formation assay

Tube formation assay was used to determine the angiogenic activity of HUVECs. Briefly, after treated with HG or H_2_O_2_, HUVECs were replated in 24-well plates that precoated with 200 μL/well growth factor-reduced matrigel (Corning, 354234) and then incubated at 37℃ in cell culture incubator. After 9 h, the capillary-like tube formation was observed under microscopy. Tube formation was defined as a tube-like structure. The tube length in duplicate wells was counted and averaged using ImageJ software.

### Cell migration assay

Briefly, HUVECs (4×10^5^/well) were seeded in six-well plates. After cultured in different low-serum medium, the cell monolayers were scratched with a sterile pipette tip to form wounds. After 6h and 24h incubation, the HUVECS were observed under a microscopy.

### ROS detection assay

DHE staining (Beyotime, S0063) or DCFH-DA (Beyotime, S0033S) was used to detect the ROS content in the spinal cord or HUVECs. DHE is oxidized by superoxide to ethidium bromide, which binds to the DNA and emits red fluorescence. The 5 μm sections of spinal cord or HUVECs were treated with 5 μM DHE reagent in a dark, then humidified chamber at 37℃ for 30 min. ROS can oxidize non-fluorescent DCFH to produce fluorescent DCF. Detection of fluorescence intensity of DCF can indicate the intracellular ROS level. HUVECs were treated with 10 μM DCFH reagent in a dark, and then humidified chamber at 37℃ for 20 min. Fluorescence intensity were observed under a Nikon ECLIPSE Ti microscope (Nikon, Japan).

### RNA sequencing and analysis

According to the instructions, the RNA was extracted from spinal cord tissue by using TRIzol reagent. The purity and quantity of RNA were identified by NanoDrop 2000 spectrophotometer (Thermo Scientific, USA). Sequencing libraries were generated using NEBNext@ Multiplex RNA Library Prep Set for llumina Novaseq 6000 (NEB, USA). The libraries were quality-assessed on an Agilent Bioanalyzer 2100 system (Agilent Technologies, USA) and sequenced on a llumina Novaseq 6000 platform. The gene expression level (FPKM) was calculated, and the reads count of each gene was obtained by HTSeq-count. The analysis of differential expression in sample (Sham *vs*. SCI; Sham *vs.* DM; Sham* vs.* DM+SCI; DM *vs.* DM+SCI; SCI *vs.* DM+SCI) was performed using the DEGseq2 package. Then, based on hypergeometric distribution algorithm, clustering analysis and KEGG enrichment analysis of different expressed genes were carried out for screening the significant enrichment.

### Detection of intracellular Ca^2+^ concentration

HUVECs were seeded at a density of 1×10^5^ cells/well in 12-well plate for 24 h. Then, HUVECs were treated with the corresponding stimuli for a period of time, and washed 3 times with a Ca^2+^-free D-Hanks balanced salt solution. Subsequently, the cells were loaded with Fluo-4AM (Beyotime, S1060) for 1 h at 37℃ in dark, then washed 2 times with Ca^2+^-free D-Hanks balanced salt solution to remove extracellular Fluo-4-AM. Therefore, the fluorescence intensity was quantitatively analyzed using a microplate reader (BioTek, USA), and qualitatively detected using a Nikon ECLIPSE Ti microscope (Nikon, Japan).

### JC-1 staining

The mitochondrial membrane potential was measured by JC-1 assay (Beyotime, C2003S). HUVECs were incubated in a mixture of culture medium and JC-1 working solution for 30 min at 37 ℃. Then, the cells were washed 3 times to remove the free JC-1 reagent. After changed with the fresh medium, the images were captured under a Nikon ECLIPSE Ti microscope (Nikon, Japan).

### Statistical analysis

All statistical analyses were performed with Graphpad prism 8.0.2. The data were presented as means ± SD. Differences between groups (for more than two groups) were analyzed by one-way ANOVA, followed by Tukey's multiple comparison test. When two variables existed, two-way ANOVA analysis was performed. P < 0.05 was considered statistically significant.

## Results

### Diabetes aggravates BSCB destruction and inhibits the recovery of locomotor function in SCI rat

**Figure 1:**
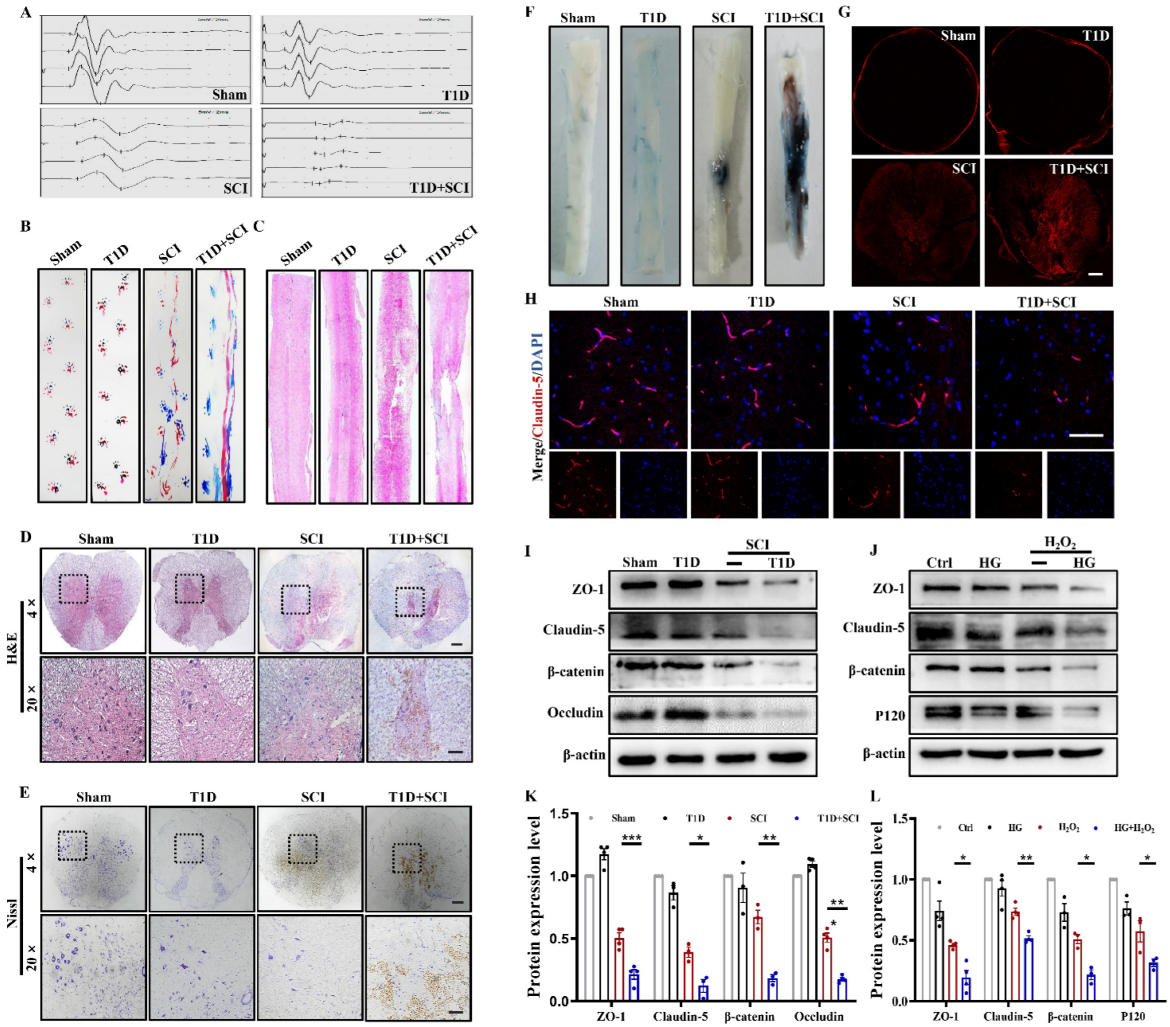
** Diabetes aggravates BSCB destruction and inhibits the recovery of locomotor function in SCI rat.** (A and B) The electromyography analysis and footprint analysis of hind limb in rat at 14 days after SCI. (C and D) Representative images of H&E staining in spinal cord of rat at 3 days after SCI, Scale bar = 500 μm (4×), Scale bar = 100 μm (20×). (E) Nissl staining of spinal cord in rat at 3 days after SCI, Scale bar = 500 μm (4×), Scale bar=100 μm (20×). (F and G) Penetration level of EB dye at 1 days after SCI, Scale bar = 500 μm. (H) The immunofluorescence image of Claudin-5 (red) level at 3 days after SCI, Scale bar = 50 μm. (I and K) Representative western blotting results and quantitative analysis of ZO-1, Claudin-5, Occludin and β-catenin in spinal cord at 3 days after SCI. (J and L) Representative western blotting results and quantification of ZO-1, Claudin-5, β-catenin and P120 in HUVECs. n≥3, *P<0.05, **P<0.01, ***P<0.001.

Here, we have confirmed the role of diabetes on locomotor function recovery after SCI. The result of electrophysiological test showed that the hindlimb of rats in T1D+SCI group exhibits lower amplitude and longer incubation when comparing with those in SCI rat (Figure 1A). The footprint analysis also revealed that the SCI rats partially recover the motor function of hindlimb at 14 dpi, while the rats in T1D+SCI group exhibit the persistent dragging hindlimb (Figure 1B). Moreover, the result of H&E staining showed that there is no significant difference of morphology in spinal cord of rat between Sham group and T1D group, but the spinal cord of SCI rat is narrowed, and has a bigger cavitation, as well as, these trends are much more serious in spinal cord of rats from T1D+SCI group (Figure 1C and 1D). Furthermore, diabetes drastically decreased the number of neurons after SCI (Figure 1E). Taken together, these results suggest that diabetes impedes the locomotor functional recovery of SCI rat.

Then, we have examined the integrity of BSCB. We found that the EB dye infiltrates into the parenchyma of spinal cord in SCI and T1D+SCI rats, but not in the spinal cord of rats from Sham and T1D group (Figure 1F and 1G). Moreover, the infiltrated EB dye was much more in spinal cord of rats from T1D+SCI group when compared to that in SCI rats (Figure 1F and 1G). These results indicate that BSCB integrity of spinal cord is severely destructed in rats from T1D+SCI group. ECs are interconnected with tight junction, and form the main barrier of BSCB 22. Here, we have tested the expressions of TJ proteins (ZO-1, claudin-5 and occludin) and AJ proteins (β-catenin and P120) *in vivo* and *in vitro*. We found that SCI significantly induces the loss of TJ proteins and AJ proteins in spinal cord of rat. And these trends were further significantly decreased in spinal cord of rats from T1D+SCI group (Figure 1H-1L).

### Diabetes significantly worsens mitochondrial dysfunction and induces excessive apoptosis of ECs in SCI rat

**Figure 2:**
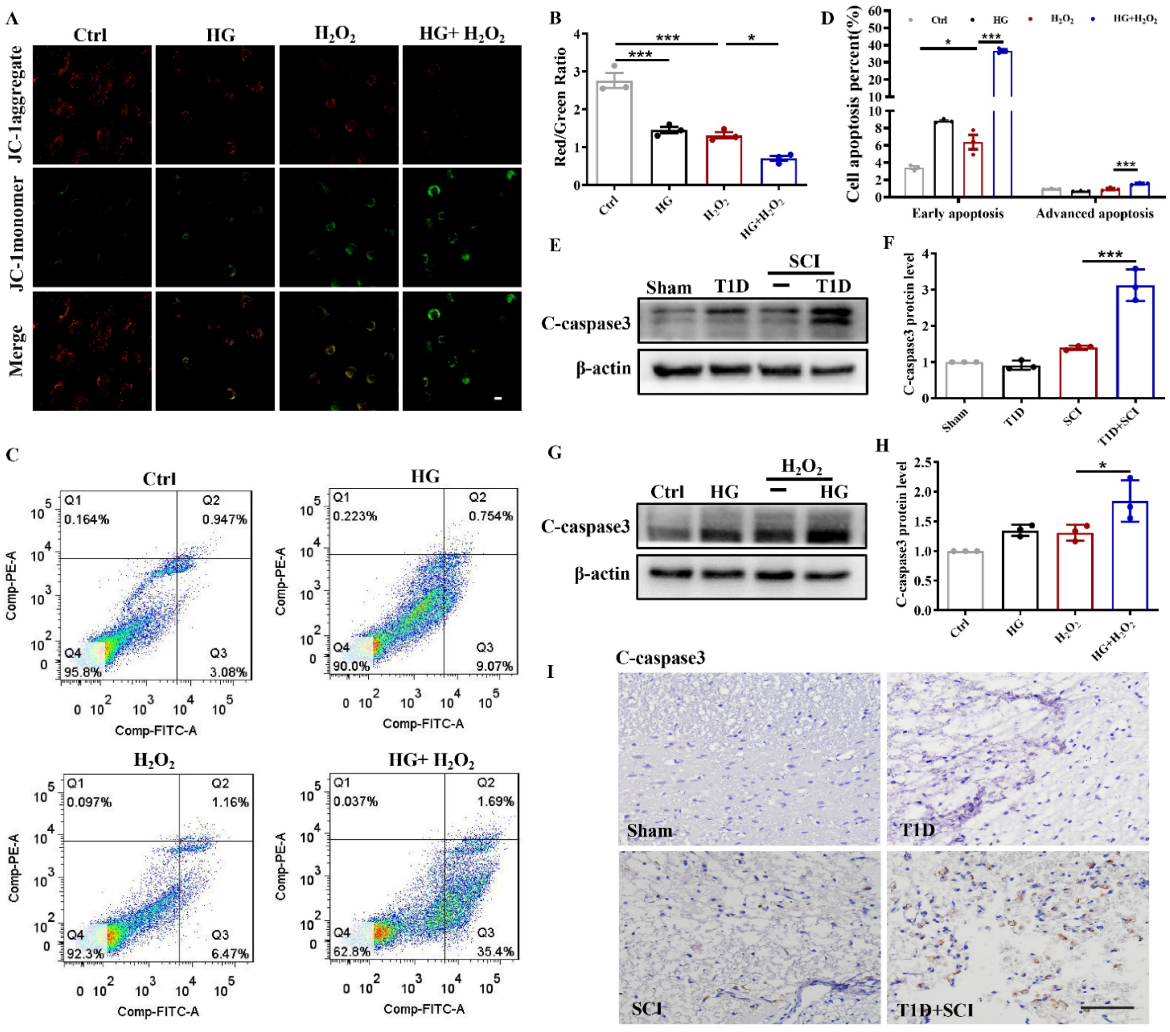
** Diabetes significantly worsens mitochondrial dysfunction and induces excessive apoptosis of ECs in SCI rat.** (A) Mitochondrial potential changes detected via JC-1 staining, Scale bar = 10 μm. (B) Quantification of Red/Green Ratio from JC-1 staining. (C) Flow cytometry examined ANXA5 in HUVECs. (D) Quantification of Flow cytometry data from (C). (E and F) Representative western blotting results and quantitative analysis of C-caspase 3 in spinal cord of rat at 3 days after SCI. (G and H) Representative western blotting results and quantitative analysis of C-caspase3 in HUVECs. (I) The immunohistochemistry staining of C-caspase 3 protein level in spinal cord of rat at 3 days after SCI, Scale bar = 50 μm. n≥3, *P<0.05, ***P<0.001.

ECs are an important component of BSCB. Using JC-1 fluorescent probe, we detected the mitochondrial membrane potential of HUVECs. In Ctrl group, JC-1 mainly existed in the mitochondrial matrix in the form of polymers, while the membrane potential was decreased after stimulated by HG or H_2_O_2_ with JC-1 existing in the cytoplasm as a monomer (Figure 2A and 2B). Membrane potential was significantly decreased or even lost after stimulated with HG+H_2_O_2_ (Figure 2A and 2B). ANNIXIN V flow cytometry analysis also showed that HG or H_2_O_2_ stimulation could significantly enhance the early apoptosis and late apoptosis of HUVECs (Figure 2C and 2D). The phenomenon of HUVECs was exacerbated when co-treating with HG+H_2_O_2_ condition (Figure 2C and 2D). To further evaluate the apoptosis level, we also performed western blotting and immunohistochemical staining for quantitative analysis of C-caspase 3 expression in spinal cord tissue. Consistent with the results of flow cytometry, the expression of C-caspase 3 was significantly enhanced in spinal cord from T1D+SCI group or HUVECs co-treated with HG+H_2_O_2_ condition when comparing with those in other groups (Figure 2E-I).

### Diabetes impedes neovascularization in spinal cord of rat after SCI

ECs participate in the formation of new blood vessels, which promotes SCI recovery. As it is known that diabetes is not conductive to angiogenesis 8, 9. Here, we have detected the role of diabetes on neovascularization in spinal cord after SCI.

**Figure 3:**
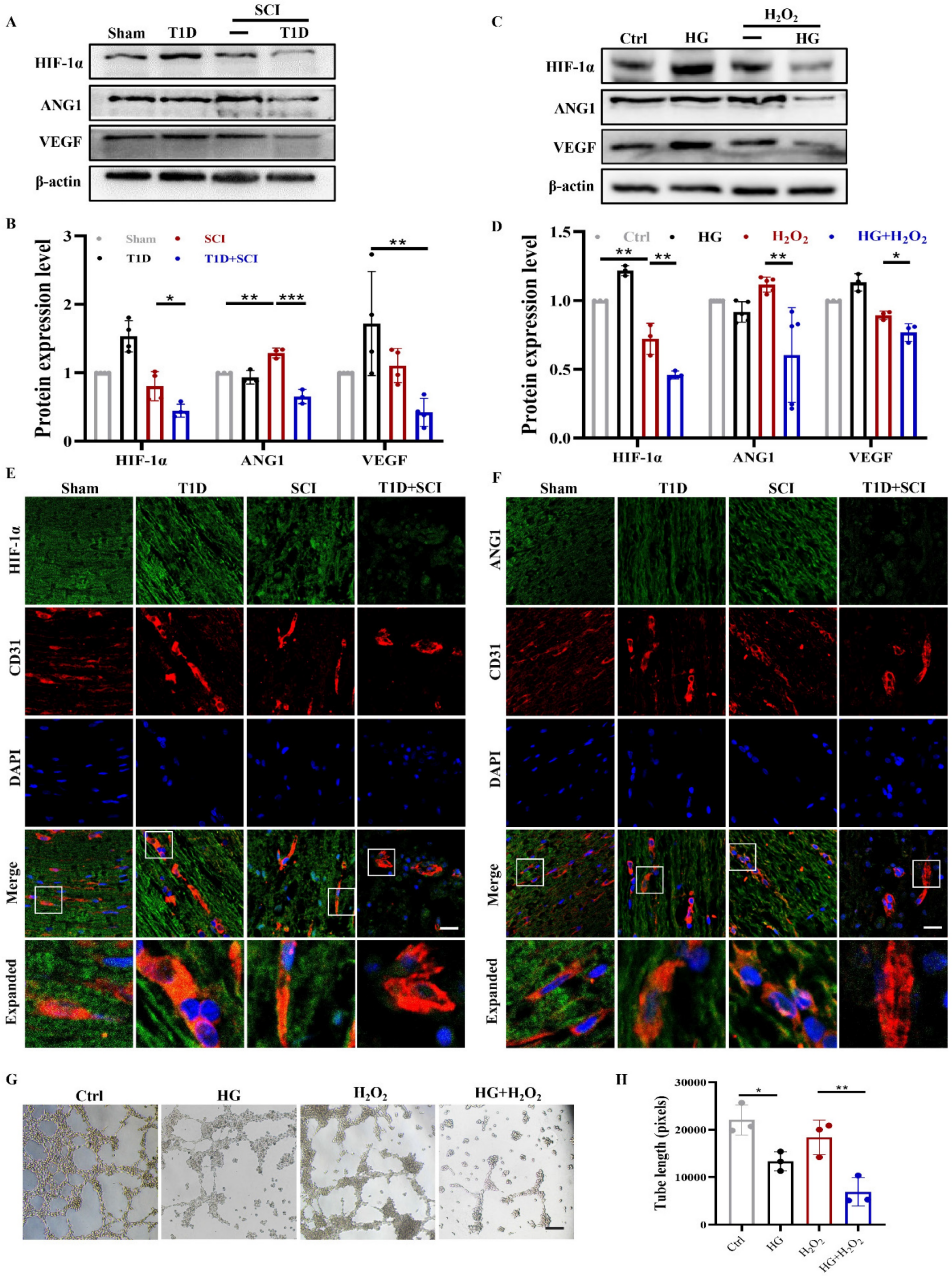
**Diabetes impedes neovascularization level in spinal cord of SCI rat.** (A and B) Representative western blotting results and quantitative analysis of HIF-1α, ANG1 and VEGF in spinal cord at 3 days after SCI. (C and D) Representative western blotting results and quantitative analysis of HIF-1α, ANG1 and VEGF in HUVECs. (E) Co-staining of HIF-1α (green) and CD31 (red) in spinal cord at 3 days after SCI, Scale bar = 10 μm. (F) Co-staining of ANG1 (green) and CD31 (red) in spinal cord at 3 days after SCI, Scale bar = 10 μm. (G) The tube formation ability of HUVECs, Scale bar = 200μm. (H) Quantification of tube length from tube formation assay. n≥3, *P<0.05, **P<0.01, ***P<0.001.

Using western blotting analysis, we found that the expressions of HIF-1α and VEGF in spinal cord from SCI rat are not significantly different when compared with those in Sham group, while ANG1 expression is significantly up-regulated, which indicates that vascular regeneration has been initiated after SCI (Figure 3A and 3B). More interesting, combined with diabetic condition, the expressions of HIF-1α, ANG1 and VEGF were all significantly decreased in spinal cord, suggesting that diabetes seriously blocks the angiogenesis in spinal cord after SCI (Figure 3A and 3B). The results *in vitro* were consistent with those *in vivo* (Figure 3C and 3D). At the same time, we further detected the expressions of HIF-1α and ANG1 in ECs of spinal cord. We had co-staining with CD31 (labeled for ECs) and HIF-1α, CD31 and ANG1. It showed that diabetes leads to significant decreases of HIF-1α and ANG1 in ECs of spinal cord after SCI (Figure S 2A, 3E and 3F). In addition, we also tested the migration and tube formation ability of HUVECs to evaluate the capacity of angiogenesis. The results showed that HUVECs have relatively strong migration and tube formation ability after H_2_O_2_ stimulation, but these ability of HUVECs were greatly decreased under co-stimulating with HG+H_2_O_2_ (Figure 3G, 3H and S 2B).

### Diabetes remarkably triggers elevated ROS and subsequent excessive oxidative stress of ECs in SCI rat

**Figure 4:**
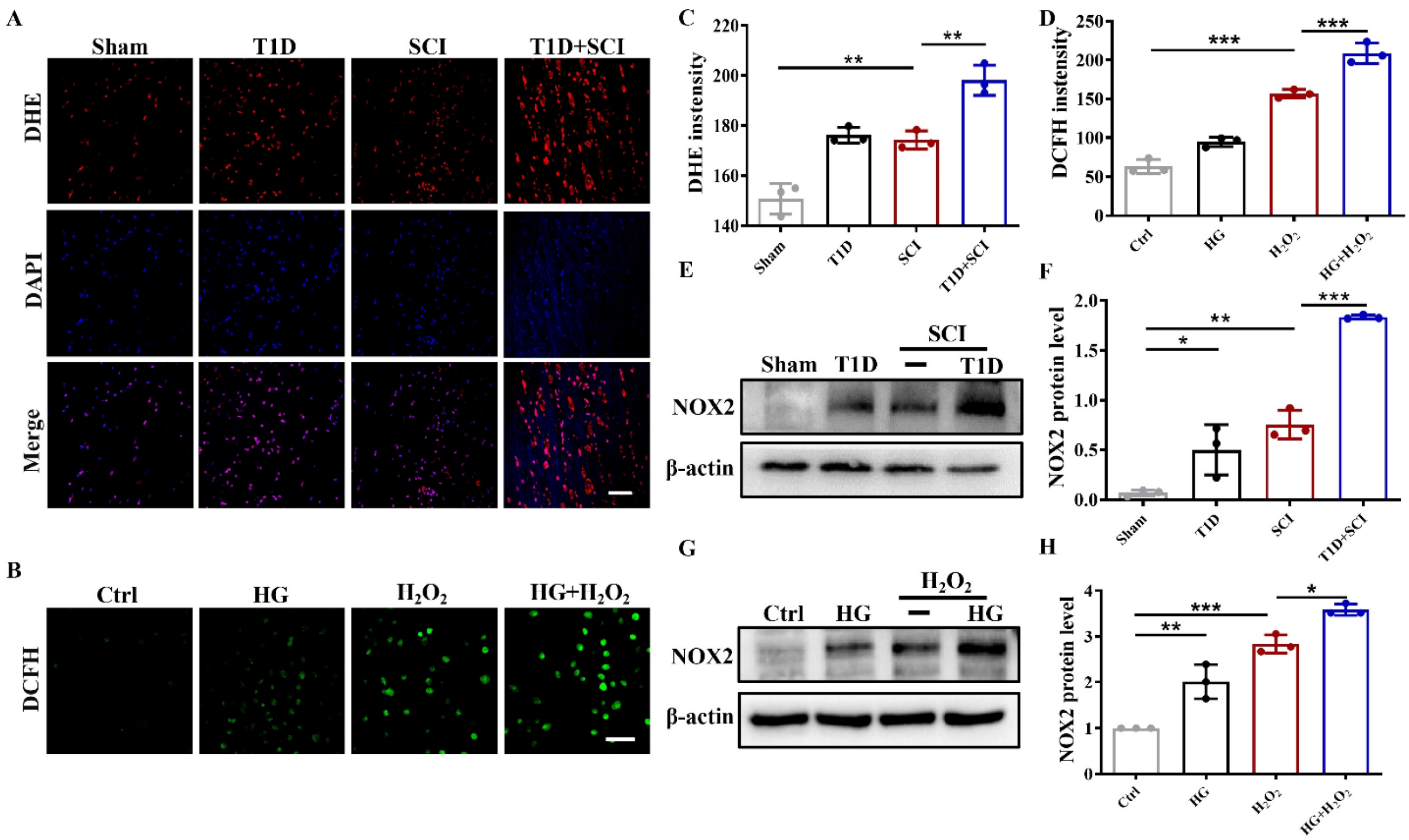
** Diabetes remarkably triggers elevated ROS and subsequent excessive oxidative stress of ECs in SCI rat.** (A and C) The DHE staining and quantitative analysis of spinal cord at 3 days after SCI, Scale bar = 50μm. (B and D) DCFH staining and quantitative analysis of HUVECs, Scale bar = 100 μm. (E and F) Representative western blotting results and quantitative analysis of NOX2 in spinal cord at 3 days after SCI. (G and H) Representative western blotting results and quantitative analysis of NOX2 in HUVECs. n≥3, *P<0.05, **P<0.01, ***P<0.001.

Oxidative stress is one of important molecular mechanism of diabetes-associated complications 23. Using DHE staining, we found that ROS level is significantly enhanced in spinal cord from SCI rat and T1D rat (Figure 4A and 4C). More importantly, SCI and diabetes has an additive effect, that is, T1D+SCI rat had much more ROS in spinal cord tissue (Figure 4A and 4C). There were similar phenomena *in vitro* (Figure 4B, 4D and Figure S 2C). NADPH oxidase (NOX) family proteins are the main cause of oxidative stress, which causes the oxidative inactivation of NO and uncoupling of eNOS, and results in the persistent oxidative stress 24. NOX has four subtypes, NOX1, NOX2, NOX4 and NOX5. Here, we found that NOX2 expression in spinal cord of rat from SCI+T1D group is significantly higher when compared with that in SCI rat (Figure 4E and 4F). The NOX2 expression in HUVECs had similar phenomena (Figure 4G and 4H). The above results indicate that diabetes induces NOX2 expression, thereby mediating the significant up-regulation of ROS in SCI rat.

### Diabetes significantly increases the expression level of TRPM2 in ECs after SCI

**Figure 5:**
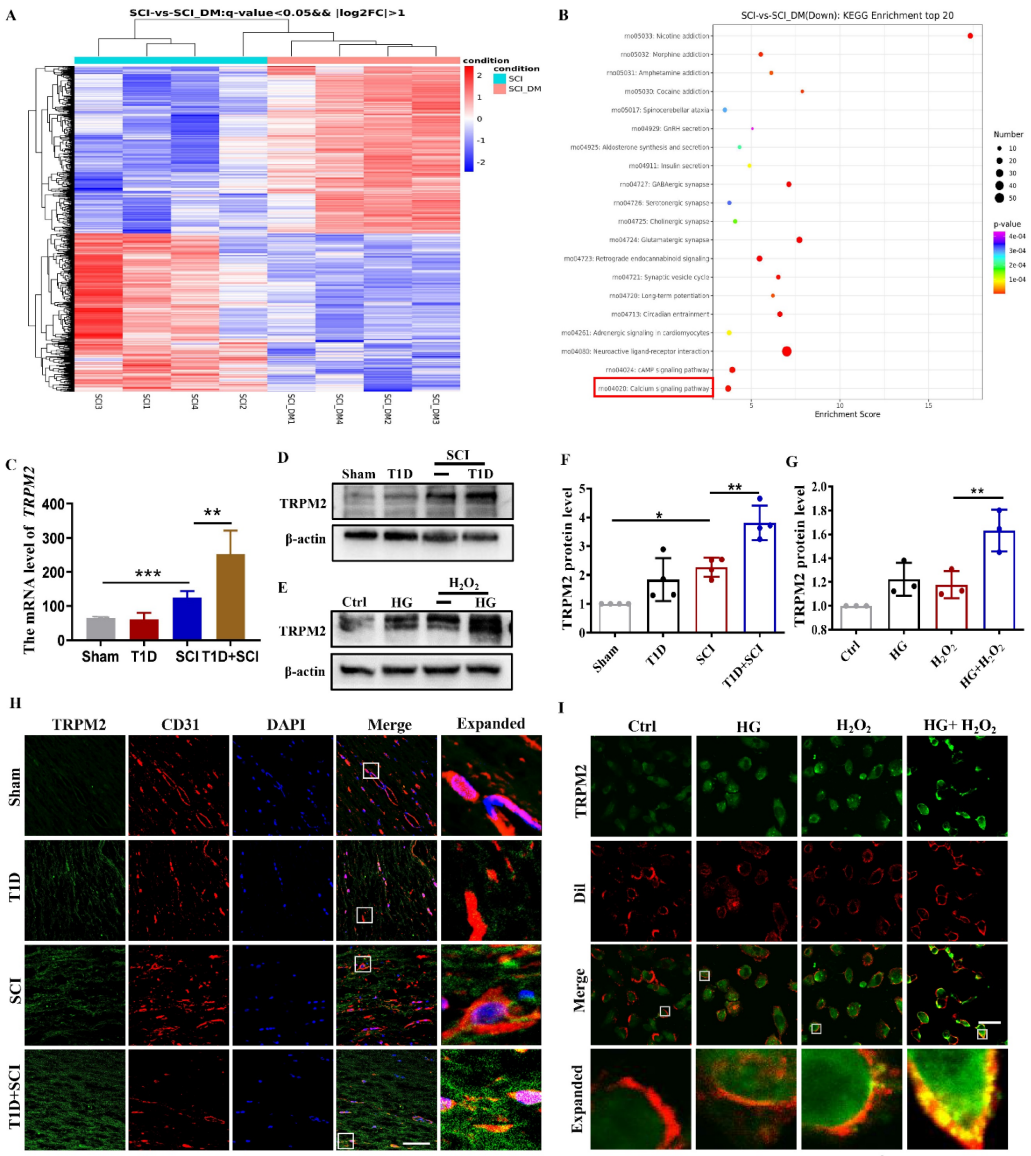
** Diabetes significantly increases the expression level of TRPM2 in ECs after SCI.** (A) The clustering analysis of gene expression based on the RNA sequencing analysis in spinal cord from SCI and DM-SCI group, n = 4. (B) KEGG enrichment analysis of gene expression based on the RNA sequencing analysis in spinal cord from SCI and DM-SCI group, n = 4. (C) The RNA level of TRPM2 in spinal cord of rat from different groups based on the RNA sequencing analysis. (D and F) Representative western blotting results and quantitative analysis of TRPM2 in spinal cord at 3 days after SCI. (E and G) Representative western blotting results and quantitative analysis of TRPM2 in HUVECs. (H) Co-staining of TRPM2 (green) and CD31 (red) in spinal cord at 3 days after SCI, Scale bar = 50 μm. (I) Co-staining of TRPM2 (green) and membrane stain Dil (red) in HUVECs, Scale bar = 50μm. n≥3, *P<0.05, **P<0.01, ***P<0.001.

As an intracellular ROS sensor, TRPM2 protein transmits information to activate Ca^2+^ influx and regulates the membrane potential, which is involved in oxidant-induced apoptosis of ECs 25-27. To explore the underlying molecular mechanism of diabetes exacerbating the BSCB destruction, we have performed the RNA sequencing (RNA-Seq) analysis, and carried out the clustering analysis and KEGG enrichment analysis. The results showed that there are series of differential expressed genes in spinal cord between DM+SCI and SCI group, especially the calcium signaling pathway, suggesting that calcium signaling pathway is significantly involved in diabetes regulating SCI recovery (Figure 5A and 5B). We have analyzed the differential expressed genes and found that comparing with SCI group, the mRNA level of *Trpm2* is significantly elevated in spinal cord from diabetes combined with SCI rat (Figure 5C). Thus, we have targeted the TRPM2 role on diabetes exacerbating the BSCB destruction. Furthermore, we had detected the expression level of TRPM2 protein in spinal cord and found that TRPM2 is up-regulated due to diabetes or SCI, more importantly, it was significantly up-regulated under diabetes combined with SCI condition (Figure 5D and 5F). To further clarify the expression pattern of TRPM2 in spinal cord tissue, we had examined the expression of TRPM2 in neuron, astrocytes and microglia and ECs respectively. It is shown that there are weak co-localization signals during co-staining of GFAP (labeled for astrocytes) or Iba1(labeled for microglia) with TRPM2 in spinal cord from different group (Figure S 2D and S 2E). During co-staining NeuN (labeled for neurons) or CD31(labeled for ECs) with TRPM2, it showed the enrichment of TRPM2 protein expression in both neurons and ECs, moreover, the expression of TRPM2 protein in ECs was significantly stronger than that in neurons in spinal cord from T1D+SCI group (Figure S 2F and Figure 5H). Moreover, the expression of TRPM2 protein in HUVECs was consistent with that *in vivo* (Figure 5E and 5G). In addition, under HG condition, H_2_O_2_ stimulation not only upregulated TRPM2 expression in cells, but also promoted the transfer of TRPM2 to membrane (Dil staining labeled for membrane) (Figure 5I). These results verify that over-expressed of TRPM2 in ECs may be the key regulatory mechanism underlying diabetes hindering SCI recovery.

### Diabetes significantly enhances TRPM2-mediated calcium influx and activates p-CaMKII/eNOS pathway in ECs after SCI

**Figure 6:**
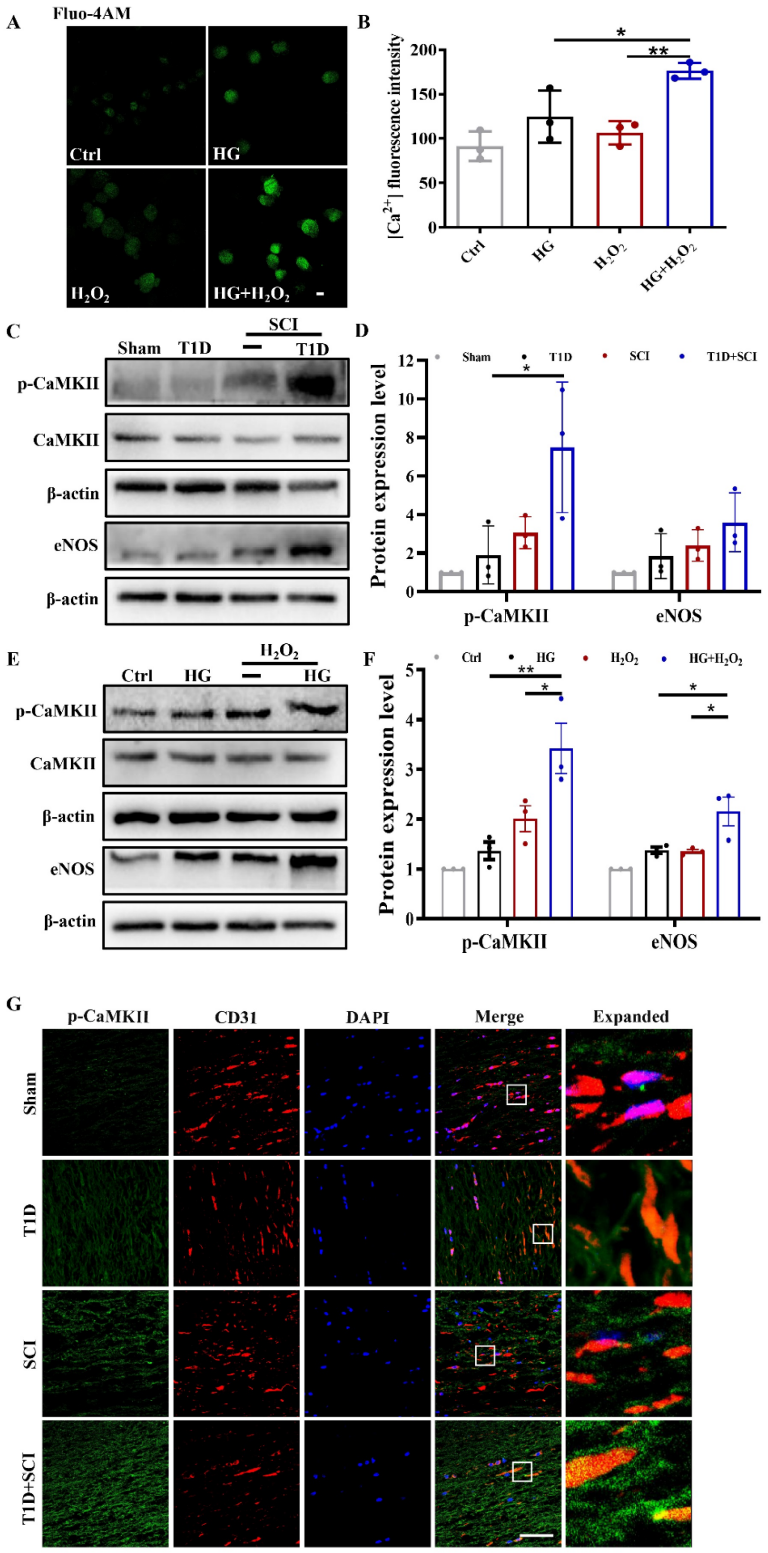
** Diabetes significantly enhances TRPM2-mediated calcium influx and activates p-CaMKII/eNOS pathway in ECs after SCI.** (A) [Ca^2+^]_i_ concentration in HUVECs via Fluo-4AM staining, Scale bar = 50 μm. (B) Quantification of fluorescence intensity of Fluo-4AM staining. (C and D) Representative western blotting results and quantitative analysis of p-CaMKII and eNOS in spinal cord at 3 days after SCI. (E and F) Representative western blotting results and quantitative analysis of p-CaMKII and eNOS in HUVECs. (G) Co-staining of p-CaMKII (green) and CD31 (red) in spinal cord at 3 days after SCI, Scale bar = 50μm. n≥3, *P<0.05, **P<0.01.

As a non-selective cation channel protein, TRPM2 can mediate Ca^2+^ influx. Ca^2+^ is an important intracellular second messenger involved in the regulation of many cellular events 28. We had used Fluo-4AM staining to analyze the [Ca^2+^]_i_ concentration in HUVECs. It was observed that [Ca^2+^]_i_ content is increased after stimulating with HG or H_2_O_2_ (Figure 6A and 6B). Moreover, Ca^2+^ influx was particularly obvious in HUVECs under co-treated with HG+H_2_O_2_ condition (Figure 6A and 6B). Elevated intracellular Ca^2+^ level will activate CaMKII, stimulate eNOS production, and which in turn increase ROS level. Ca^2+^/CaMKII pathway plays an important role in the development of diabetic vascular dysfunction, including diabetic heart disease 29. In current study, it was found that T1D+SCI and HG+H_2_O_2_ both enhance the expressions of p-CaMKII and its downstream signal of eNOS following the increased Ca^2+^ influx *in vivo* and vitro (Figure 6C-6F). The result of immunofluorescence also showed that the expression of p-CaMKII in ECs is significantly enhanced in the spinal cord from T1D+SCI group, moreover, the number of new blood vessels is obviously decreased when compared with those in SCI rat (Figure 6G). These results indicate that TRPM2-mediated Ca^2+^ influx significantly activates CaMKII in spinal cord of SCI rat under diabetic environment, and then further upregulates eNOS, which is not conducive to angiogenesis during SCI recovery.

### TRPM2 inhibition reverses the adverse effect of diabetes on locomotor function recovery in SCI rat

**Figure 7:**
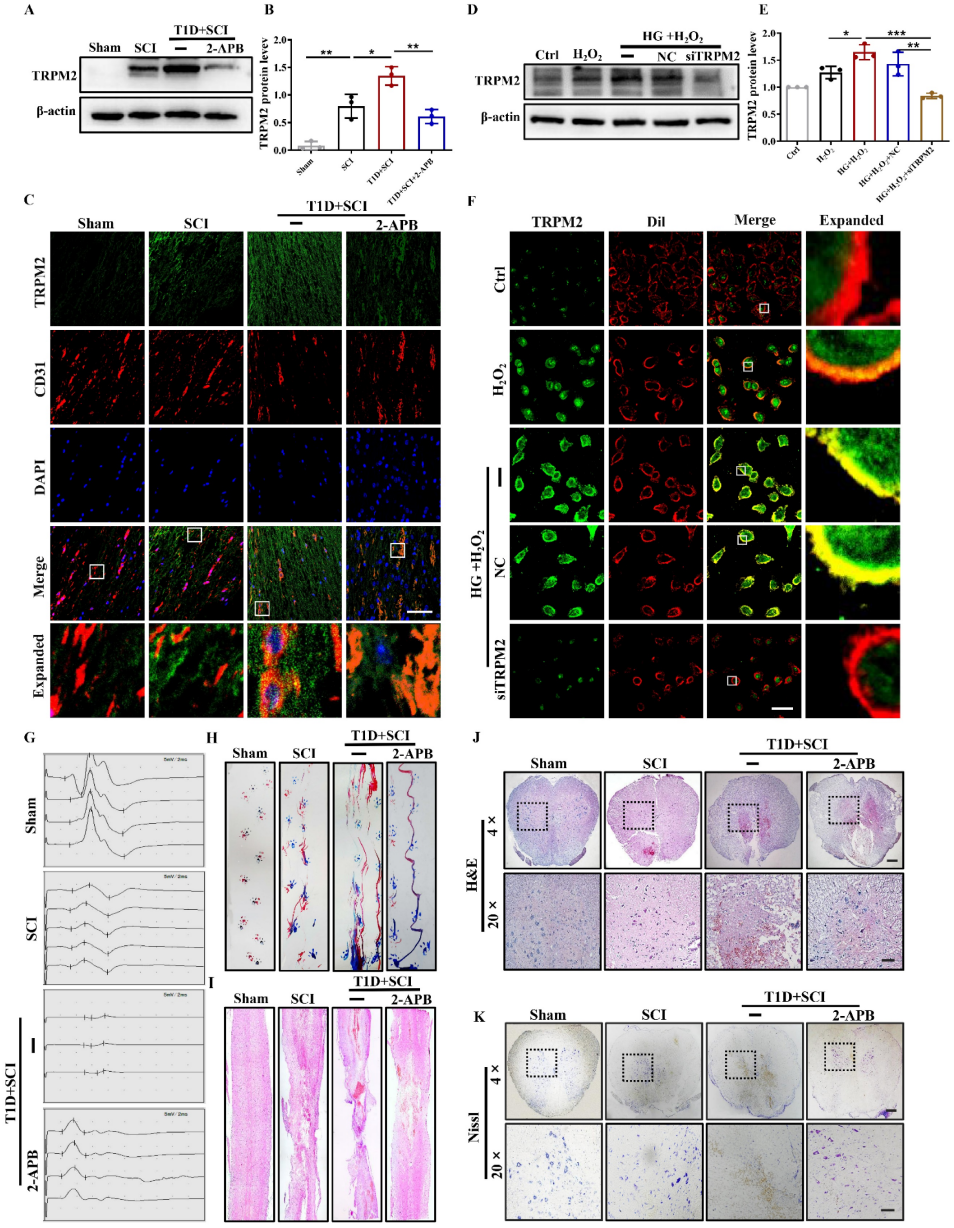
** TRPM2 inhibition reverses the effect of diabetes on locomotor function in SCI rat.** (A and B) Representative western blotting result and quantitative analysis of TRPM2 in spinal cord at 3 days after SCI. (C) Co-staining of TRPM2 (green) and CD31 (red) in spinal cord at 3 days after SCI, Scale bar = 50 μm. (D and E) Representative western blotting result and quantitative analysis of TRPM2 in HUVECs. (F) Co-staining of TRPM2 (green) and membrane stain Dil (red) in HUVECs, Scale bar = 50 μm. (G and H) The electromyography analysis and footprint analysis of hind limb in rat at 14 days after SCI. (I and J) Representative images of H&E in spinal cord of rat at 3 days after SCI, Scale bar = 500 μm (4×), Scale bar = 100 μm (20×). (K) Nissl staining of spinal cord of rat at 3 days after SCI, Scale bar = 500 μm (4×), Scale bar = 100 μm (20×). n≥3, *P<0.05, **P<0.01 and ***P<0.001.

Then, we used TRPM2 inhibitor (2-APB) and TRPM2 siRNA to validate the role of TRPM2 on BSCB integrity. It was observed that 2-APB treatment significantly decreases TRPM2 protein level in the spinal cord from T1D+SCI group (Figure 7A and 7B). Moreover, the results of co-staining CD31 with TRPM2 showed that expression level of TRPM2 on ECs from T1D+SCI rat is significantly decreased after treating with 2-APB, moreover, the number of new blood vessels is increased (Figure 7C). *In vitro*, the expression level and membrane translocation of TRPM2 in HUVECs from HG+H_2_O_2_ group was also significantly inhibited by TRPM2 siRNA treatment (Figure 7D-7F).

Next, we analyzed the effect of 2-APB treatment on locomotor function of rat from T1D+SCI group. The electrophysiological result showed that 2-APB treatment not only reduces the incubation period of hindlimb in the rat from T1D+SCI group, but also increases the amplitude of muscle (Figure 7G). In addition, the footprint analysis also indicated that 2-APB treatment partially promotes the locomotor function recovery of hindlimb in T1D+SCI group with no longer exhibiting persistent hindlimb dragging (Figure 7H). Furthermore, the 2-APB treatment reduced the cavitation area of injury site and loss of neuron in the spinal cord from T1D+SCI group (Figure 7I-7K). These results suggest that TRPM2 inhibition contributes to the recovery of locomotor function in diabetes combined with SCI rat.

### TRPM2 inhibition partly restores BSCB integrity in diabetes combined with SCI rat

**Figure 8:**
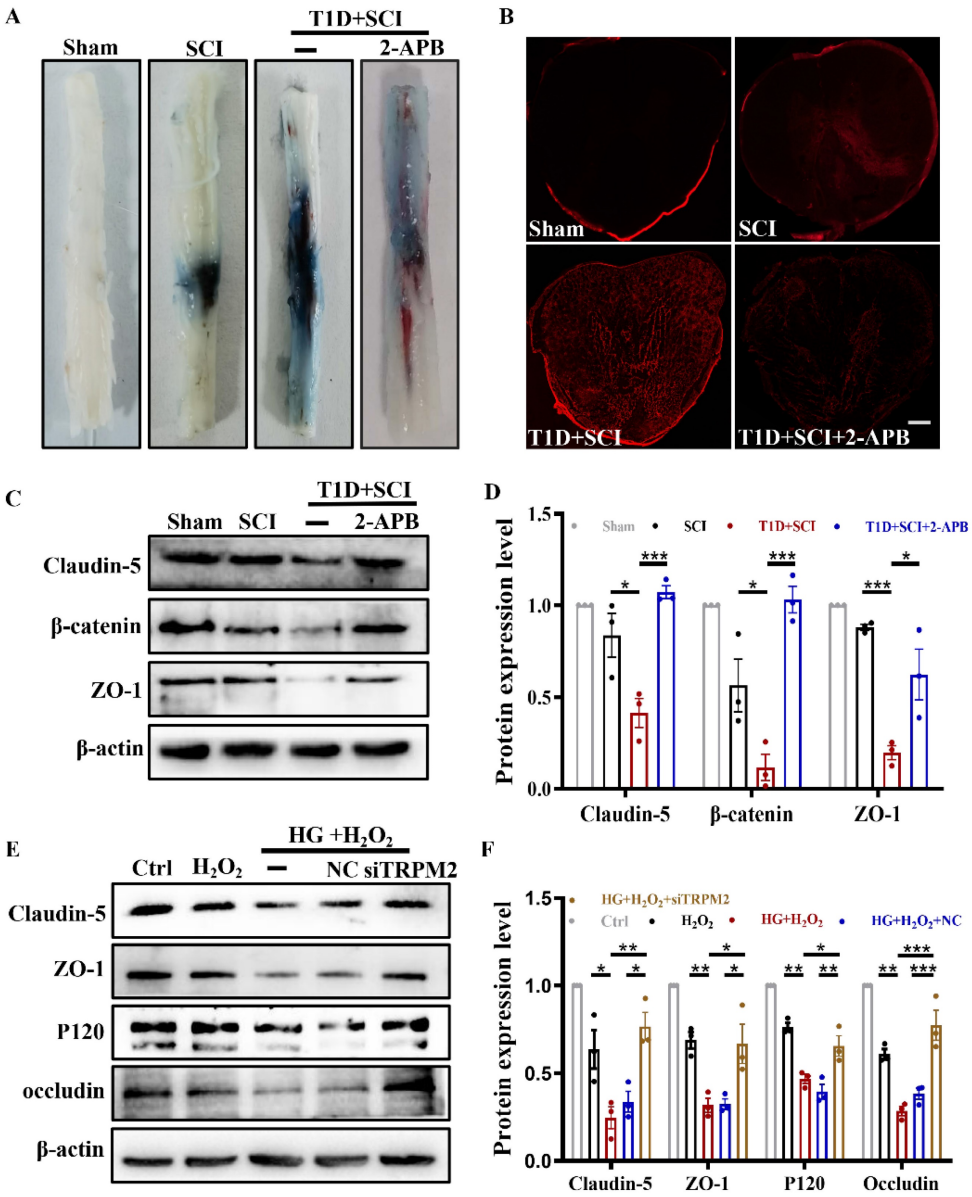
** TRPM2 inhibition restores BSCB integrity in diabetes combined with SCI rat.** (A and B) The penetration of EB dye in spinal cord of rat at 1 days after SCI, Scale bar = 500 μm. (C and D) Representative western blotting results and quantitative analysis of Claudin-5, β-catenin and ZO-1 in spinal cord of rat at 3 days after SCI. (E and F) Representative western blotting results and quantitative analysis of Claudin-5, ZO-1, P120 and occludin in HUVECs. n≥3, *P<0.05, **P<0.01, ***P<0.001.

Here, we have further examined whether TRPM2 inhibition is beneficial to restore BSCB integrity. As it is shown that comparing with T1D+SCI group, 2-APB treatment reduces the EB dye penetration and weakens the fluorescence intensity of EB dye in spinal cord from T1D+SCI rat (Figure 8A and 8B). Moreover, the expression levels of Claudin-5, ZO-1 and β-catenin in spinal cord of T1D + SCI rat were significantly increased after treating with 2-APB (Figure 8C and 8D). TRPM2 siRNA also enhanced the expressions of these proteins in HUVECs under HG+H_2_O_2_ condition (Figure 8E and 8F). Taken together, TRPM2 inhibition partially restores BSCB integrity in spinal cord from diabetes combined with SCI rat, indicated that TRPM2 is the key target for diabetes exacerbating BSCB destruction.

### TRPM2 inhibition effectively reduces the ROS level of ECs through suppressing CaMKII/eNOS signaling

**Figure 9:**
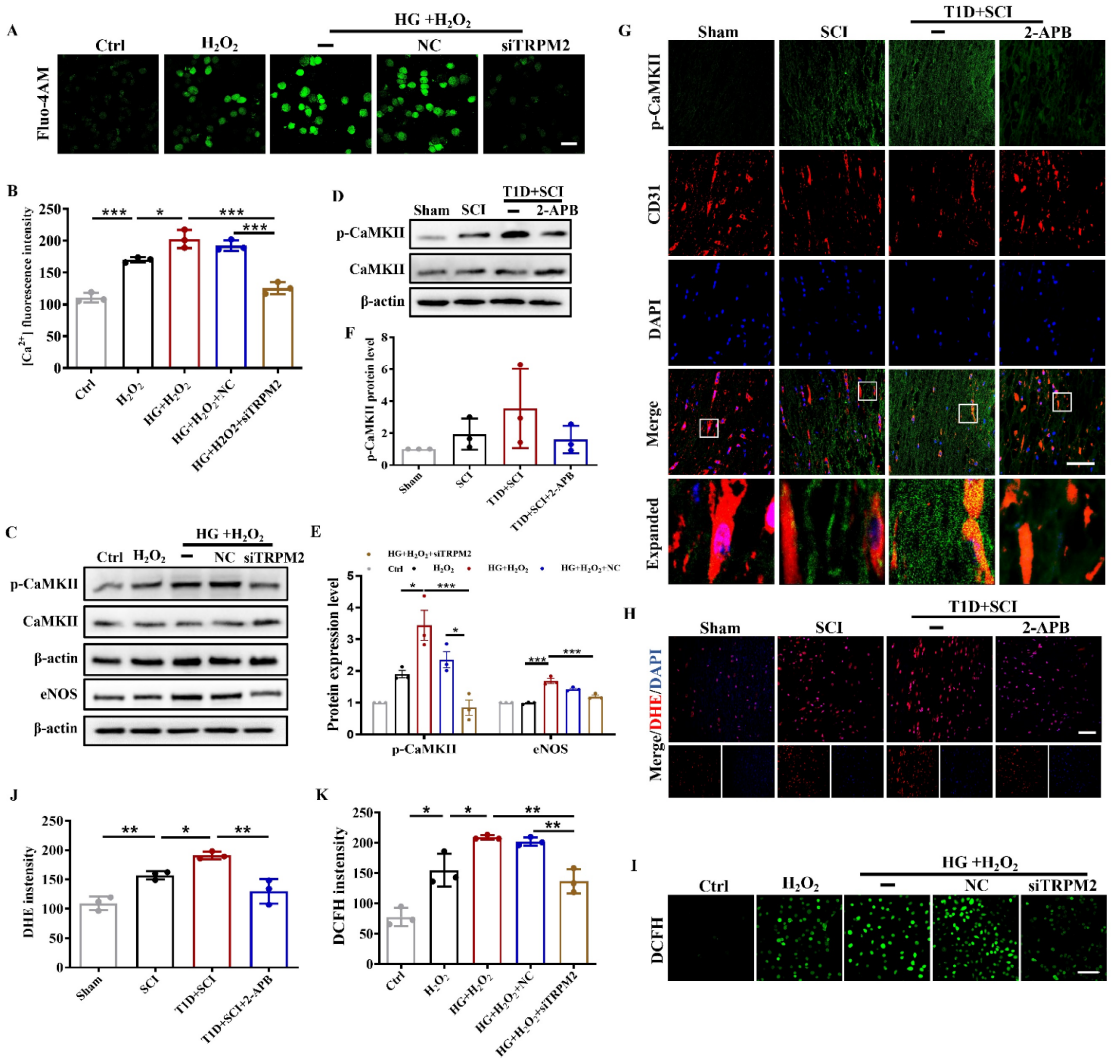
** Inhibition TRPM2 effectively reduces ROS level of ECs through suppressing CaMKII/eNOS signaling.** (A and B) Fluo-4AM staining and quantitative analysis of [Ca^2+^]_i_ concentration in HUVECs, Scale bar = 50 μm. (C and E) Representative western blotting results and quantitative analysis of p-CaMKII and eNOS in HUVECs. (D and F) Representative western blotting result and quantitative analysis of p-CaMKII in spinal cord of rat at 3 days after SCI. (G) Co-staining of p-CaMKII (green) and CD31 (red) in spinal cord of rat at 3 days after SCI, Scale bar = 50 μm. (H and J) The image and quantitative analysis of DHE staining in spinal cord at 3 days after SCI, Scale bar = 50 μm. (I and K) The image and quantitative analysis of DCFH staining in HUVECs, Scale bar = 100 μm. n≥3, *P<0.05, **P<0.01, ***P<0.001.

Next, we quantitative analysis the role of TRPM2 inhibition on [Ca^2+^]_i_ content *in vitro*. In the HUVECs from HG+H_2_O_2_ group, we found [Ca^2+^]_i_ concentration was significantly higher than that in H_2_O_2_ group (Figure 9A and 9B). However, TRPM2 siRNA treatment significantly inhibited Ca^2+^ influx in the HUVECs from HG+H_2_O_2_ group (Figure 9A and 9B). Subsequently, we further detected the expression levels of p-CaMKII and its downstream factor of eNOS *in vivo* and *in vitro*.

The results showed that the expressions of p-CaMKII and eNOS are suppressed when TRPM2 in spinal cord or HUVECs is inhibited by 2-APB or TRPM2 siRNA treatment (Figure 9C-9F). Furthermore, we confirmed that 2-APB treatment inhibits the level of p-CaMKII in ECs from T1D+SCI group (Figure 9G). Subsequently, we observed that 2-APB treatment or TRPM2 siRNA reverses the elevated ROS level in spinal cord from T1D+SCI rat or HUVECs in HG+H_2_O_2_ condition (Figure 9H-9K and Figure S 2G). These results suggest that TRPM2 effectively induces the elevated ROS level in ECs through activating CaMKII/eNOS signaling.

### TRPM2 inhibition effectively ameliorates mitochondrial dysfunction and excessive apoptosis of ECs in diabetes combined with SCI rat

**Figure 10:**
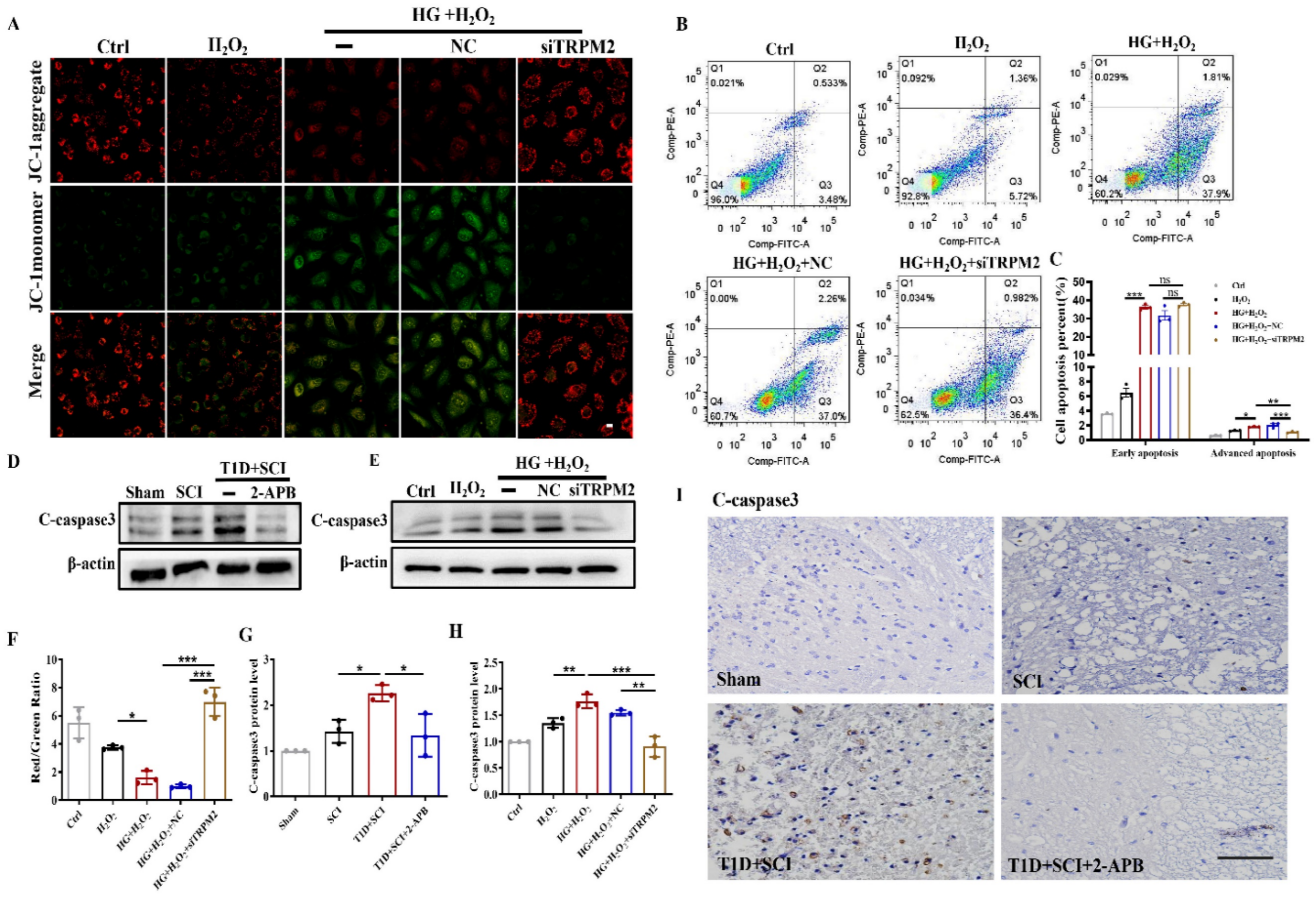
** TRPM2 inhibition effectively ameliorates mitochondrial dysfunction and excessive apoptosis of ECs in diabetes combined with SCI rat.** (A) Mitochondrial potential changes detected via JC-1 staining, Scale bar = 10 μm. (B) Flow cytometry examined ANXA5 in HUVECs. (C) Quantitative analysis of Flow cytometry data from (B). (D and G) Representative western blotting result and quantitative analysis of C-caspase3 in spinal cord of rat at 3 days after SCI. (E and H) Representative western blotting result and quantitative analysis of C-caspase3 in HUVECs. (F) Quantification of Red/Green Ratio of (A). (I) The immunohistochemistry staining of C-caspase3 expression in spinal cord of rat at 3 days after SCI, Scale bar = 50 μm. n≥3, *P<0.05, **P<0.01, ***P<0.001.

To verify the regulatory role of TRPM2-mediated Ca^2+^ overload on mitochondrial function, we also examined the mitochondrial ΔΨm potential and apoptosis level of ECs after TRPM2 inhibition. The results showed that TRPM2 siRNA could significantly restore the mitochondrial ΔΨm potential to a level close to the normal state (Figure 10A and 10F). Moreover, although TRPM2 siRNA treatment had no protective effect on early apoptosis induced by HG+H_2_O_2_ stimulation, it could reduce the proportion of late apoptotic cells (Figure 10B and 10C). However, the expression level of C-caspase3 was significantly decreased in spinal cord from T1D+SCI+2-APB group or HUVECs in HG+H_2_O_2_+TRPM2 siRNA condition (Figure 10D, 10E, 10G-10I).

### TRPM2 inhibition improves angiogenesis level under diabetes combined with SCI condition

**Figure 11:**
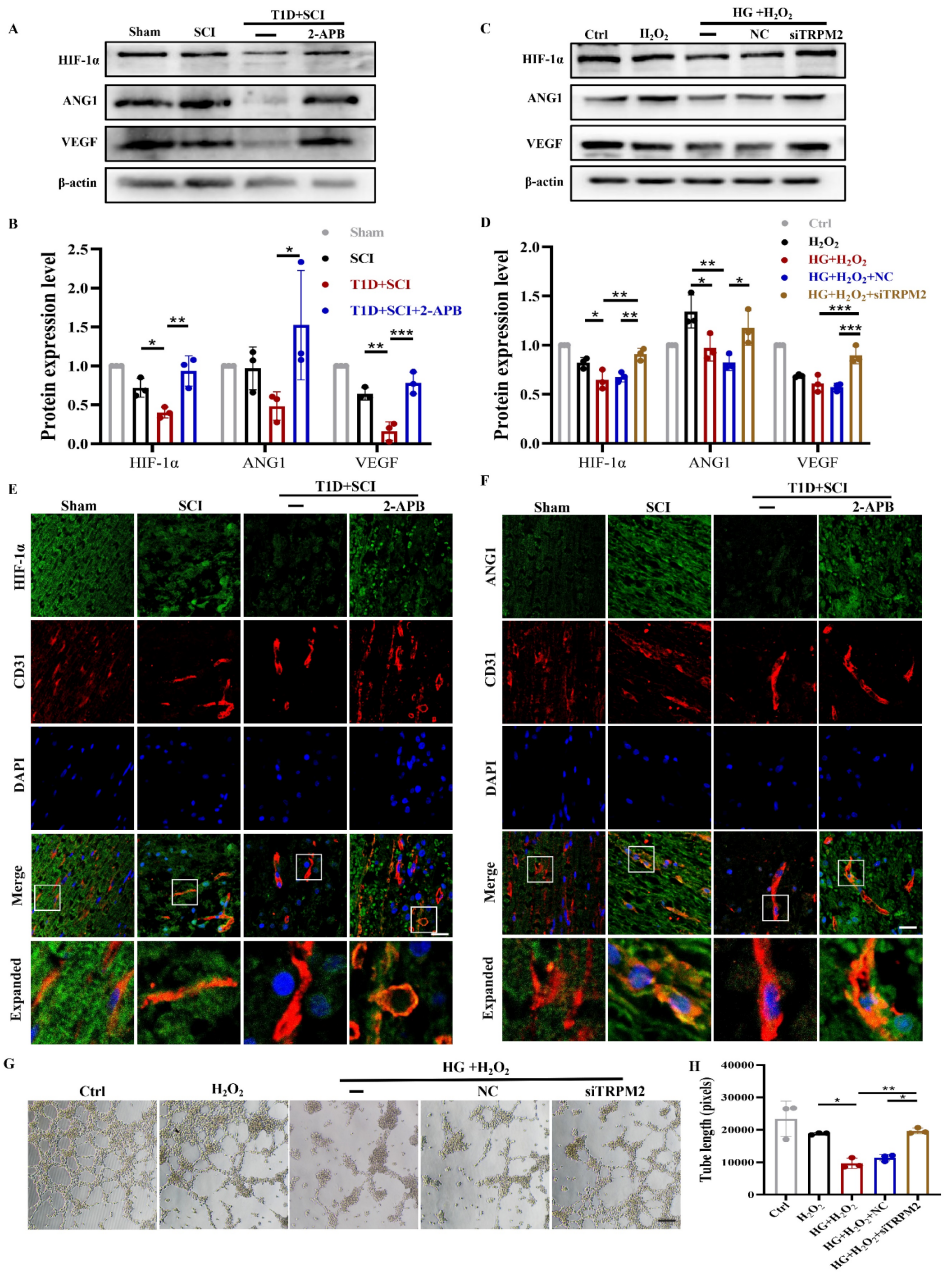
** TRPM2 inhibition improves angiogenesis level under diabetes combined with SCI condition.** (A and B) Representative western blotting results and quantitative analysis of HIF-1α, ANG1 and VEGF in spinal cord of rat at 3 days after SCI. (C and D) Representative western blotting results and quantitative analysis of HIF-1α, ANG1 and VEGF in HUVECs. (E) Co-staining of HIF-1α (green) and CD31 (red) in spinal cord at 3 days after SCI, Scale bar = 10 μm. (F) Co-staining of ANG1 (green) and CD31 (red) in spinal cord at 3 days after SCI, Scale bar = 10 μm. (G) The tube formation ability of HUVECs, Scale bar = 200 μm. (H) Quantification of tube length from tube formation assay. n≥3, *P<0.05, **P<0.01, ***P<0.001.

We have confirmed that both 2-APB and TRPM2 siRNA treatment could inhibit CaMKII/eNOS signaling in ECs. Here, we have further examined the effect of TRPM2 inhibition on angiogenesis ability during SCI recovery. The results showed that 2-APB treatment could significantly increase the protein levels of HIF-1α, ANG1 and VEGF in spinal cord from T1D+SCI group, which contributed to angiogenesis to some extent (Figure 11A and 11B, Figure S 2H). Moreover, we have also confirmed it *in vitro* (Figure 11C and 11D). We have co-stained CD31 (labeled for ECs) with HIF-1α and ANG1, and found that the expressions of ANG1 and HIF-1α in ECs of spinal cord from T1D+SCI group are significantly enhanced after 2-APB treatment (Figure 11E and 11F). The migration and tube formation ability of HUVECs in HG+H_2_O_2_ group were recovered by TRPM2 siRNA treatment, and which reached a state similar to those in H_2_O_2_ group (Figure 11G and 11H and Figure S 2I). These results indicate that TRPM2 inhibition contributes to the angiogenesis in spinal cord during SCI recovery.

## Discussion

SCI is an important pathogenic factor of disability in young adults. Except for primary injury, SCI will induce secondary injury, which triggers oxidative stress and inflammation, and lastly results in difficulty of axon and myelin regeneration 3, 4. SCI seriously decreases the living quality of patients. Diabetes is detrimental to SCI repair 7. Our current study has confirmed that diabetes significantly aggravates SCI and is not conducive to the repair of BSCB integrity. Diabetes is a systemic metabolic disorder. Thus, the influence of diabetes on SCI repair is extremely complicated and multi-factorial. Diabetes is reported to increase the apoptosis of ECs after SCI 11. However, the molecular mechanism under it is still unclear. In current study, we focused on TRPM2 ion channel, and revealed that diabetes triggers TRPM2 over-expression and activates TRPM2 ion channel, which will induce excessive apoptosis of ECs and aggravate BSCB destruction.

Ion channels is closely related to nerve injury repair 30. After CNS injury, the regular changes of calcium channel-related protein expression and electrical activity are closely related to the inflammatory reaction in the early stage of injury and the change of [Ca^2+^]_i_, which participates in the nerve injury repair 31, 32. TRPM2 ion channel is a kind of intracellular ADP-ribose (ADPR)-gated non-selective Ca^2+^ ion channel 33.

In this study, we observed that diabetes significantly enhances the expression of TRPM2 in ECs after SCI. 2-APB was used to inhibit the expression of TRPM2. It was showed that TRPM2 inhibition alleviates the adverse effect of diabetes on apoptosis and angiogenesis in ECs of spinal cord, consequently promotes the locomotor function recovery of diabetes combined with SCI rat. These results suggest that TRPM2 protein may be an important target for diabetes hindering BSCB repair after SCI. Of course, TRPM2 ion channel maybe not the only regulatory channel of Ca^2+^. As it is well known that Piezo protein is also an important Ca^2+^ ion channel. Therefore, the regulatory effect of diabetes on other ion channels and the relationship between TRPM2 channel and other ion channels are need to be further explored in future.

TRPM2 ion channel is one of key molecular mechanism of oxidative stress-associated diseases 21. It has been showed that alcohol induces activation of TRPM2 ion channel through NOX/ROS/PARP signaling pathway, and then promotes microglia death 34. In addition, oxidative stress activates ER stress and promotes the pericyte death by mediating activation of TRPM2 channel 21. Oxidative stress is also an important molecular mechanism of diabetes-associated complication 35. We found that diabetes also significantly induces the oxidative stress level in the spinal cord after SCI. More importantly, we found that after SCI, excessive ROS may be an important mechanism for diabetes promoting the over-expression of TRPM2 on ECs. It was observed that TRPM2 inhibitor (2-APB) treatment significantly suppresses ROS level in spinal cord from diabetes combined with SCI rat. These results suggest that TRPM2-mediated ion channel maybe an important agonist for elevated oxidative stress injury in ECs under diabetic condition. Activity of TRPM2 ion channel and ROS are synergistic and mutually reinforcing processes in ECs during diabetes aggravating SCI.

The disorderd cellular microenvironment is one of key factors that hinders the recovery of nerve function after SCI. After SCI, the cellular microenvironment has dramatically changed under BSCB destruction. As we all known, BSCB destruction leads to the poor blood supply of injury area in spinal cord, and results in the necrosis of spinal cord tissues. Additionally, peripheral circulating proteins and inflammatory factors uncontrollably enter the injured area, and aggravate the inflammatory response and hypoxia ischemia, which further reduces the pH value in the injured area and forms a local acidic environment 36. Thus, after SCI, protection of BSCB integrity and promoting angiogenesis in the injured area play a vital role in the survival of nerve cells and maintenance of nerve function. In our current study, we found that except for the apoptosis of ECs in spinal cord, diabetes could also induce the adverse microenvironment for angiogenesis with significantly inhibiting expressions of HIF-1α, VEGF, and ANG1 in spinal cord after SCI. 2-APB treatment increased the number of blood vessels in spinal cord tissue from diabetes combined with SCI group. This result suggests that TRPM2 may also be an important target for maintenance of the microenvironment for SCI recovery under diabetic condition.

ECs dysfunction is the earliest and most fundamental pathological change of diabetes. A large amount of evidence has shown that hyperglycemia mediates excessive superoxide induces the injury of vascular cells 12, 13. Programmed cell death (PCD) requires three stages: early apoptosis, advanced apoptosis and end apoptosis. Inhibition of TRPM2 may have a protective effect on apoptosis by alleviating DNA damage. Considering that a large number of cells were in the early apoptotic stage after stimulation, but did not enter the advanced apoptotic stage, the detection results showed only a weak protective effect on apoptosis. Hyperglycemia induces a large amount of O_2_^•-^ through the electron transport chain of mitochondrial, while over-produced superoxide will enhance the following pathways, including protein kinase C, glycosylation end products and hexosamine pathways, which induces DNA damage and thus causes the damage of vascular cells 37. This explains that diabetes alone also promotes oxidative stress, TRPM2 over-expression and ECs apoptosis in spinal cord tissue. But overall, the effect of diabetes alone on oxidative stress or TRPM2 expression is more similar to those in SCI alone. However, diabetes alone has little effect on locomotor function of SCI rat, which should be attributed to the hyperglycemia duration of action. Here, the duration of hyperglycemia on nerve cells in spinal cord was 2 weeks, which may be far from the directly affecting neurological function. In general, the dual factors of diabetes combined with SCI have more serious effect on ECs and the microenvironment of angiogenesis after injury.

**Figure 12:**
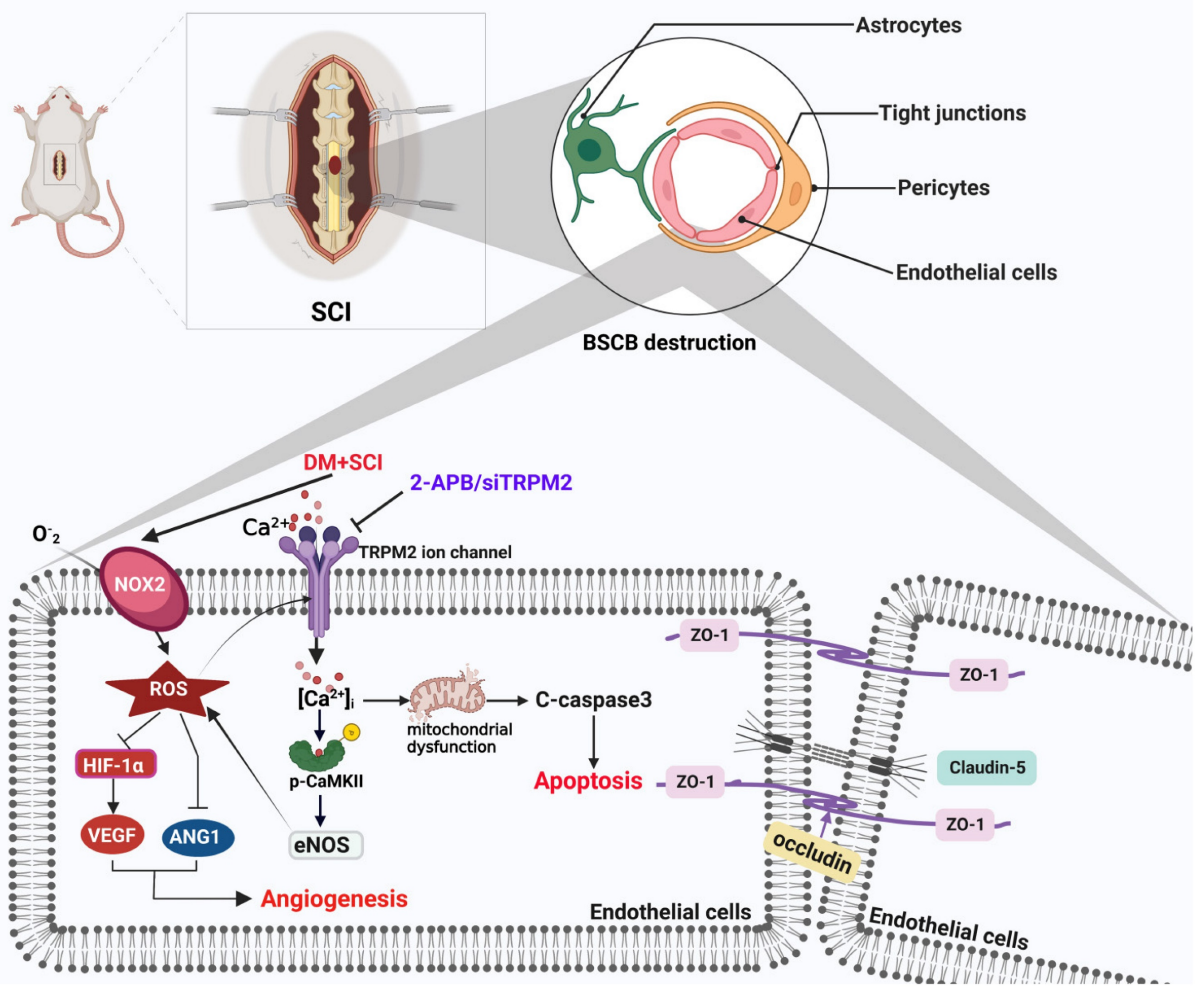
** Schematic diagram of the regulatory role of TRPM2 ion channel on BSCB integrity in diabetes combined with SCI rat.** Under diabetic condition, TRPM2 mediates Ca^2+^ influx to activate CaMKII after SCI. Then, p-CaMKII further upregulates eNOS level and promotes ROS production, which forms a positive feedback regulation of ROS. Subsequently, diabetes-medicated elevated ROS level has triggered excessive apoptosis of ECs and inhibited angiogenesis, and lastly aggravates BSCB destruction and impedes SCI recovery.

In this study, we confirmed that diabetes is not conducive to the repair of BSCB destruction after SCI, and further revealed that diabetes will mediate Ca^2+^ influx by inducing the over-expression of TRPM2 in ECs and activating TRPM2 ion channel (Figure 12). Ca^2+^ overload not only induces the mitochondrial dysfunction, thereby promoting the apoptosis of ECs, but also triggers the over-expression of eNOS by activating CaMKII, consequently promotes ROS production, which is detrimental for angiogenesis. However, TRPM2 expression is also enriched in neurons. Thus, a further investigate into the effect of neuron-derived TRPM2 protein on diabetes combined with SCI recovery is needed. In a conclusion, this study has partly revealed the regulatory role of the TRPM2 ion channel on diabetes combined with SCI recovery, and will provide a potential target for the repair of diabetes complicated with SCI.

## Supplementary Material

Supplementary figures.

## Abbreviations

T1D: Type 1 diabetes mellitus
BSCB: Blood spinal cord barrier
[Ca^2+^]_i_: Intracellular free calcium ion concentration
2-APB: 2-Aminoethyl diphenylborinate
ROS: Reactive oxygen species
SCI: Spinal cord injury
HIF-1α: Hypoxia inducible factor-1α
VEGF: Vascular endothelial growth factor
ANG: 1Angiopoietin 1
TRP: Transient receptor potential
TRPM2: Transient receptor potential melastatin 2
HUVECs: Human umbilical vein endothelial cells
ECs: Endothelial cells
TJ: Tight junction
AJ: Adhesion junction
