# Supplementary material for: Over-activation of TRPM2 ion channel accelerates blood-spinal cord barrier destruction in diabetes combined with spinal cord injury rat
Source: Int J Biol Sci. 2023 May 8;19(8):2475–94. doi: 10.7150/ijbs.80672 (PMC10197895; doi:10.7150/ijbs.80672)

**Supplementary Figure 1.** (A) Timeline diagram of diabetes combined with spinal cord injury, drug treatment, and experimental analysis in rats. (B) Experimental design in vitro. (C) The body weight of rats after injected with STZ. (D) The blood glucose level of rats after injected with STZ. (E) The blood glucose level of rats after SCI. (F) Cell viability of HUVECs under different concentration of glucose (G) Cell viability of HUVECs under treatment with mannitol or high glucose. (H) Cell viability of HUVECs under different concentration of $H_2O_2$.

**Supplementary Figure 2.** (A and H) The immunohistochemistry staining of HIF-1α and ANG1 protein level in spinal cord of rat at 3 days after SCI, Scale bar = 100 μm. (B and I) The migration ability of HUVECs at 0 h, 6 h and 24 h, Scale bar = 500 μm. (C and G) The images of DHE staining in HUVECs, Scale bar = 50 μm. (D) Co-staining of TRPM2 (green) and GFAP (red) in spinal cord at 3 days after SCI, Scale bar = 50 μm. (E) Co-staining of TRPM2 (green) and Iba1 (red) in spinal cord at 3 days after SCI, Scale bar = 50 μm. (F) Co-staining of TRPM2 (green) and NeuN (red) in spinal cord at 3 days after SCI, Scale bar = 50 μm.

## Supplementary Figure 1

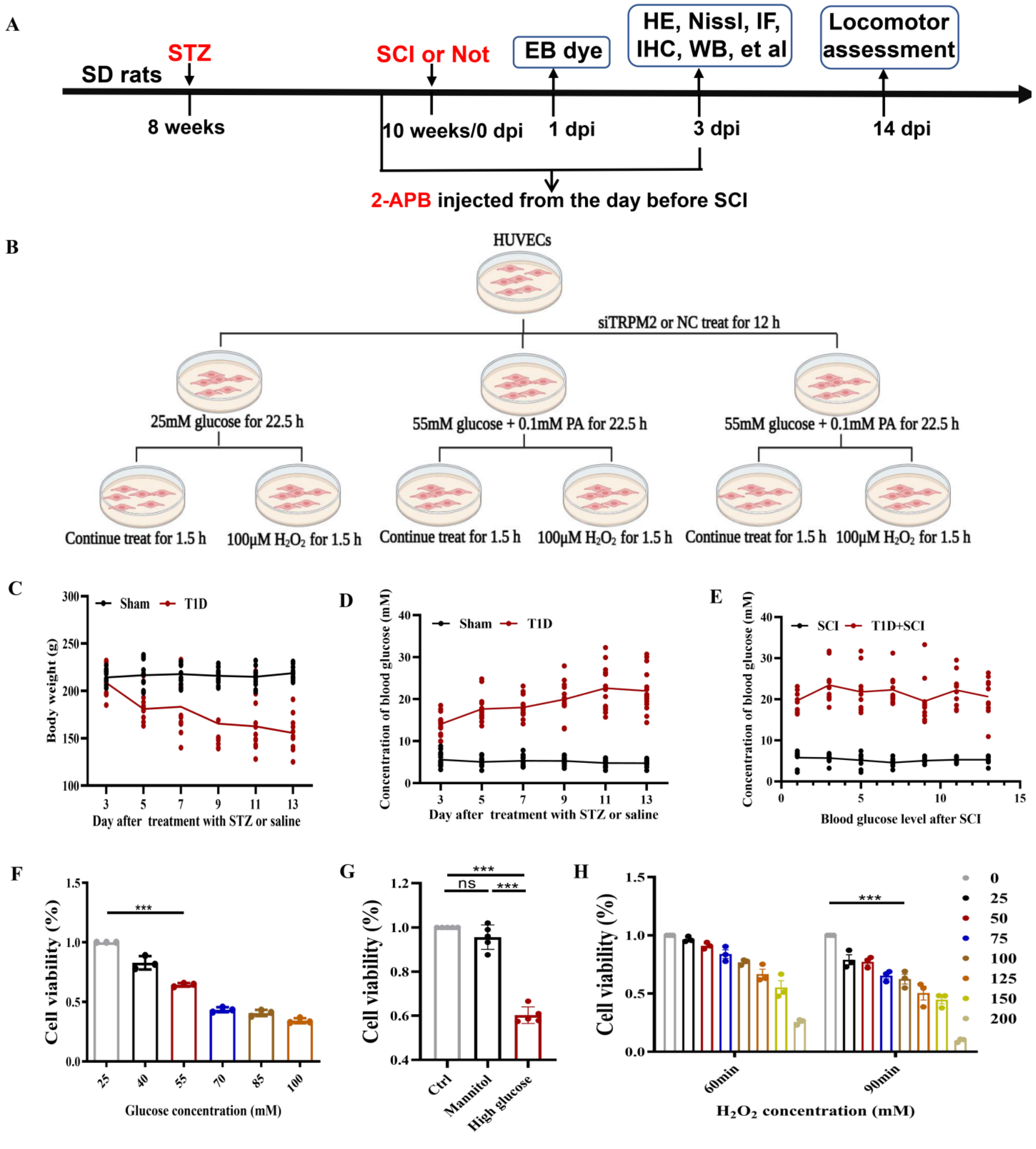

# Supplementary Figure 2

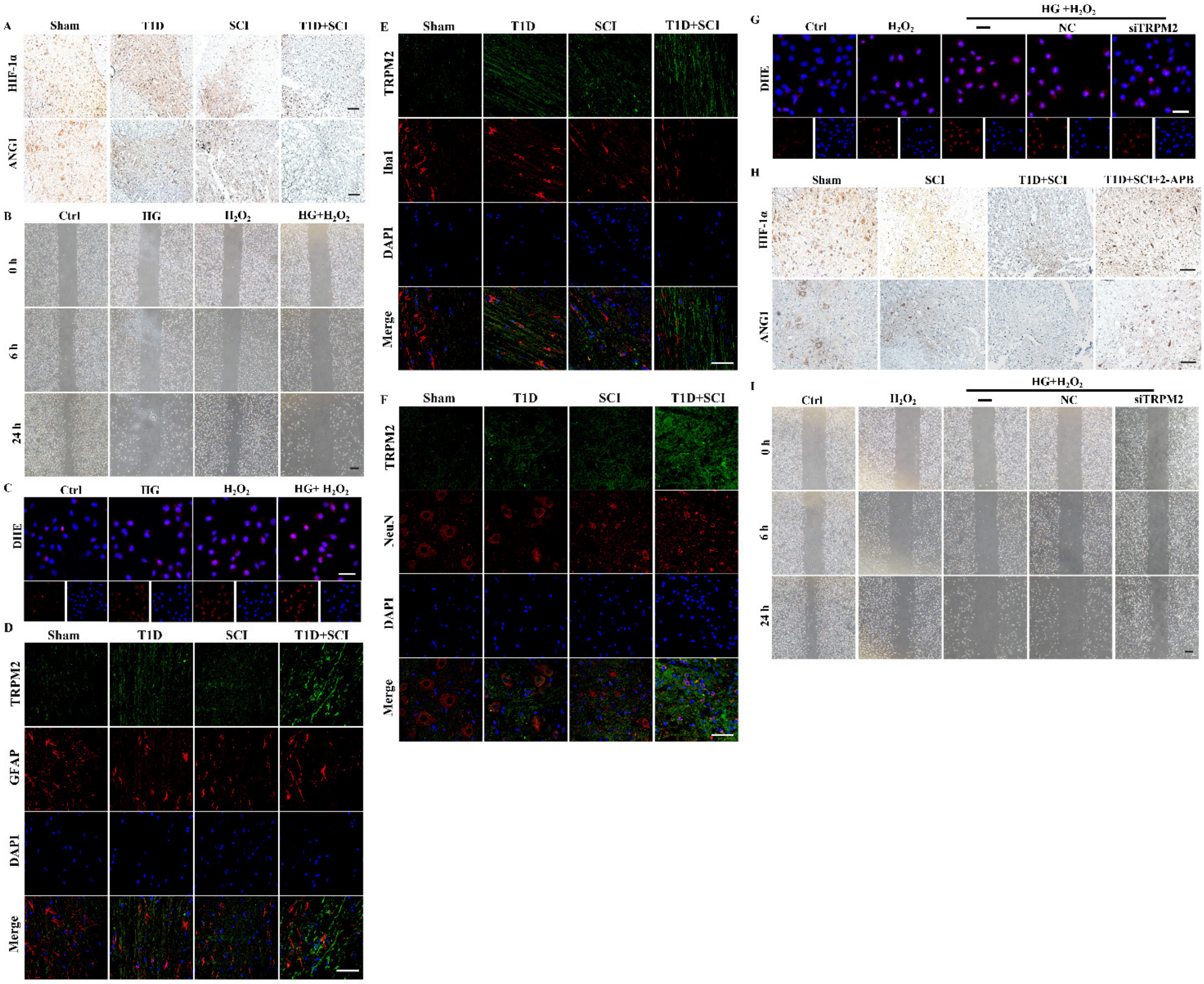

Supplement: Supplementary file 1 — Supplementary figures. [file ijbsv19p2475s1.pdf]
